# Distribution bias analysis of germline and somatic single-nucleotide variations that impact protein functional site and neighboring amino acids

**DOI:** 10.1038/srep42169

**Published:** 2017-02-08

**Authors:** Yang Pan, Cheng Yan, Yu Hu, Yu Fan, Qing Pan, Quan Wan, John Torcivia-Rodriguez, Raja Mazumder

**Affiliations:** 1 The Department of Biochemistry & Molecular Medicine, The George Washington University Medical Center, Washington, DC 20037, United States of America; 2 The Department of Statistics, The George Washington University, Washington, DC 20037, United States of America; 3 McCormick Genomic and Proteomic Center, The George Washington University, Washington, DC 20037, United States of America

## Abstract

Single nucleotide variations (SNVs) can result in loss or gain of protein functional sites. We analyzed the effects of SNVs on enzyme active sites, ligand binding sites, and various types of post translational modification (PTM) sites. We found that, for most types of protein functional sites, the SNV pattern differs between germline and somatic mutations as well as between synonymous and non-synonymous mutations. From a total of 51,138 protein functional site affecting SNVs (pfsSNVs), a pan-cancer analysis revealed 142 somatic pfsSNVs in five or more cancer types. By leveraging patient information for somatic pfsSNVs, we identified 17 loss of functional site SNVs and 60 gain of functional site SNVs which are significantly enriched in patients with specific cancer types. Of the key pfsSNVs identified in our analysis above, we highlight 132 key pfsSNVs within 17 genes that are found in well-established cancer associated gene lists. For illustrating how key pfsSNVs can be prioritized further, we provide a use case where we performed survival analysis showing that a loss of phosphorylation site pfsSNV at position 105 in MEF2A is significantly associated with decreased pancreatic cancer patient survival rate. These 132 pfsSNVs can be used in developing genetic testing pipelines.

With the advancement of high-throughput sequencing (HTS) technology, the cost of sequencing the human genome has dropped significantly^1^,^2^. However, while many biologists expected that genome sequencing could solve human health issues in a short period of time, complex diseases, such as cancer, still remain difficult to tackle^3^. In the field of cancer genomics, several international collaborations, such as The Cancer Genome Atlas (TCGA) (http://cancergenome.nih.gov/), International Cancer Genome Consortium (ICGC)^4^, have provided useful HTS based genomics data by sequencing a large number of tumor samples across cancer types^5^,^6^,^7^. The availability of large number of samples across different types of cancer enables pan-cancer analysis which explores via comparative analysis various cancer genomes originating from different tumor types^8^,^9^. By investigating the similarities and differences of cancer genomes and cellular characteristics across cancer types, tumor heterogeneity has been better understood^10^,^11^ and a number of cancer associated pathways and genes have been identified^7^,^12^,^13^,^14^. Furthermore, such analysis can reveal how mutations affect protein function. Our previous study^8^ shows the landscape of protein functional site affecting non-synonymous single-nucleotide variations (nsSNVs) across cancer types. In the current study we extensively investigate the abundance or depletion of SNV (both synonymous and non-synonymous) occurrence in different protein functional site type and the immediate region of the protein functional site. We also perform a comparative study on the SNV occurrence between germline and somatic mutations impacting different functional sites. Previous studies show that synonymous mutations are not always silent and they are able to cause changes in protein expression, conformation and function^15^,^16^,^17^,^18^,^19^. Therefore, we also compare the frequencies of synonymous and non-synonymous mutations on protein functional sites.

Since proteins are the foundational and functional blocks of living organisms, how genomic alterations of protein coding genes affect protein functionality is an important question. While many previous publications have focused on genes through pan-cancer analysis, our efforts extend the utility of a pan-cancer analysis by examining the effect of genomic alterations on protein functional sites. To this end, we have retrieved a comprehensive collection of SNVs and protein functional sites, including post-translational modification (PTM), ligand binding site, and enzyme active site, from a variety of data sources. Somatic mutations were retrieved from COSMIC^20^, UniProtKB^21^, TCGA (http://cancergenome.nih.gov/), and ICGC^4^. Germline mutations were retrieved from dbSNP^22^. All SNVs were unified and mapped to amino acid positions. To facilitate the pan-cancer analysis, the original annotated cancer types retrieved from source databases were mapped to Disease Ontology (DO) slim terms^23^. Protein functional sites were retrieved from UniProKB sequence feature (FT) line^21^, NCBI Conserved Domain Database (CDD)^24^, and dbPTM^25^. By integrating SNVs and protein functional sites, we can identify functional site affecting SNVs (pfsSNVs) for downstream analysis.

In this study, we first obtained a global perspective on how germline and somatic mutations are distributed at the proteome level, especially on various protein functional sites through integrating 3,342,377 SNVs (1,501,666 germline mutations and 1,840,711 somatic mutations) and 268,478 known and curated PTM sites, binding sites and enzyme active sites. Then we created a framework to facilitate this SNV prioritization process using observed frequency in patients and cancer type information.

## Materials and Methods

### SNV dataset

**Figure 1:**
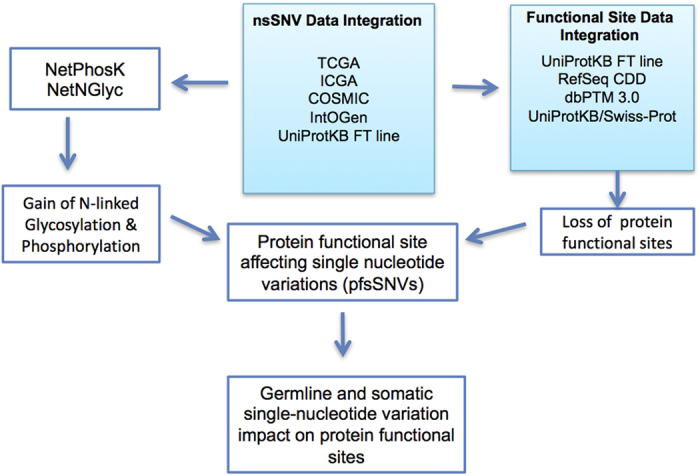
Flowchart of the distribution bias analysis of protein functional site affecting single nucleotide variations (pfsSNVs).

As the flowchart in Fig. 1 shows, somatic coding mutations were extracted from ICGC (version v0.10a), TCGA (release January 27, 2015), COSMIC (version v73), IntOGen (release 2014.12), and ClinVar (release 20150205). All somatic mutations were unified and then annotated using ANNOVAR^26^. Cancer types were mapped to DO Cancer Slim terms^23^ for cancer term unification. Frequency of a certain mutation was either calculated based on patient ID or was directly extracted from the downloaded files. All integrated information is stored and can be downloaded from the BioMuta database^8^. SNVs annotated as the same variation but from different sources/patients were collapsed into a single entry, but all relevant source information was maintained.

Germline coding mutations were collected from dbSNP (build 142) database. Minor Allele Frequency (MAF) and “Common/Rare SNP” tags were directly extracted from dbSNP. All SNVs were translated and mapped to the UniProtKB complete human proteome set (downloaded in January 2015) through a pairwise-alignment based pipeline for unification and downstream protein functional site analysis.

### Protein functional site dataset

Protein post-translational modification (PTM), binding, and enzyme active site annotation were extracted from three different sources: dbPTM 3.0^25^, UniProtKB/Swiss-Prot feature (FT) line (January 2015), and CDD features (January 2015). Only experimentally verified data were retrieved from dbPTM 3.0 and UniProtKB. Duplicates and conflicted accessions were removed. Variants with the same annotation from different sources were collapsed into a single data point while maintaining source information. Modification data was extracted using PTMlist, a controlled vocabulary provided by UniProtKB/Swiss-Prot. The NCBI CDD-based annotation of functional sites was retrieved using BATCH CD-Search against CDART database^27^. Entries such as domains, repeats, and motifs with longer than five consecutive amino acids were not considered. Filtered sites were categorized manually into various types of PTM sites, active sites, and binding sites with original annotations maintained in a separate column. Other PTM records were adopted based on dbPTM 3.0 which collects PTM data from more than 10 different sources^25^.

All entries were unified based on the UniProtKB complete human proteome set downloaded from UniProtKB on January 2015, which is identical to the proteome used for SNVs dataset unification.

### Mapping SNVs to protein functional sites and the neighboring positions

The general process of mapping SNVs to protein functional sites includes loading the SNV file into matrix of “UniProt accession with UniProt Position” and match it to the protein functional site matrix. Once the protein accession and position are matched, additional steps were used to evaluate if this SNV caused a substitution at the functional site or not. If the SNV is a substitution, we also consider the known amino acid tolerance for corresponding PTM type, if the substitution replaces the original residue with a residue which cannot be modified as a PTM or function as an active site. The output file provides a tab-delimited file containing all SNVs and affected protein functional site information. A SNV ratio based on SNV numbers divided by proteome length was calculated for expected SNV number as well as the statistical significance using methods described earlier^8^. The SNV occurrence between protein functional site and all other amino acid located within +/− 20 amino acids was compared and the significance was evaluated through one sample *t*-test.

### SNV-caused gain of protein phosphorylation and glycosylation site prediction

NetNGlyc (v1.0) and NetPhosK (v1.0) were used to predict SNV-caused gain of protein phosphorylation and N-glycosylation site^28^,^29^. 21 mer and 5 mer were set as the effective segment length of input sequences for phosphorylation and glycosylation site prediction respectively. For parameters, ESS filter and threshold 0.6 were applied for NetPhosK, while a score 0.6 is required for NetNGlyc prediction result. Both protein reference sequence and mutated sequence were used as input to the NetNGyc and NetPhos in order to minimize false positives by subtracting background predicted sites.

### Statistical significance of amino acid based pfsSNV occurrence

To investigate whether the distinct frequency of SNV on protein functional sites is caused by different amino acid mutation rate, we conducted amino acid based binomial test on pfsSNV occurrence.

First, for each type of amino acid (denote as *A*), we first calculate the probability of *A* to be a *F* type of protein functional site, calculated as following:


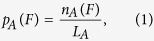


where *L*_*A*_ denotes total number of amino acid *A* on human proteome, *n*_*A*_(*F*) denotes the total number of positions for a specific functional site with amino acid *A*. Thus, amino acid based protein functional site rate *p*_*A*_(*F*) can be derived from our protein functional site dataset.

Then, we calculated the expected number of pfsSNVs *n*_*A*_(*E*) for each type of amino acid:


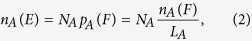


where *N*_*A*_ is total number of variations with amino acid type *A. n*_*A*_(*E*) is then used to derive if the given type of pfsSNV occurrence on the given amino acid type A is enriched or depleted.

Next, after obtaining from our SNV dataset the value of observed pfsSNV *n*_*A*_(*O*) for a specific *A* and *F*, the binomial test was performed according to Mi *et al*.^30^, and the p-value was calculated as the total probabilities to observe *n*_*A*_ the same as or more extreme (larger if *n*_*A*_(*O*) is larger than expected and smaller otherwise) than *n*_*A*_(*O*), which measures the deviance degree between an expected ratio (*n*_*A*_(*E*)/*N*_*A*_ or *p*_*A*_(*F*)) and an observed ratio (*n*_*A*_(*O*)/*N*_*A*_):


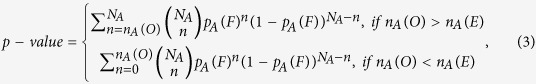


Comparing to our previous study where the same expected SNV rate applying to all protein functional site^8^, advantage of this background SNV rate is that this allows each type of protein functional site having different expected SNV rates given different components of amino acid as their donor site.

### Pan-cancer clustering of pfsSNV profiles

In order to investigate the somatic pfsSNV occurrence pattern in each cancer type, a pan-cancer analysis was performed. The observed and expected somatic mutation occurrence among each cancer type among different protein functional site type was calculated following same rule described under ‘Mapping SNVs to protein functional sites and the neighboring positions’. Basically the observed value is the mutation occurrence on a type of protein functional site while expected value is the average of neighboring mutation occurrence. And the fold change was used as a metric to perform hierarchical clustering (HC). The heat map was generated via the R package *ggplot* version 2.17.0^31^.

### pfsSNVs prioritization criteria

Two distinct criteria were used to prioritize pfsSNV: a) pfsSNVs that exist across 5 or more cancer types, b) pfsSNVs that are enriched in patients with certain cancers. To do this we leveraged TCGA patient counts mapped to our mutation dataset to identify key pfsSNVs. We combined pfsSNVs that can cause either a loss or gain of functional site. The Binomial test described above (section “Statistical significance of amino acid based pfsSNV occurrence”) was applied to identify pfsSNVs that is significantly associated with a certain cancer type based on enrichment in patients with that cancer. In this calculation, we calculated the expected probability of any type of pfsSNV occurring in a patient in a cancer type *C*:


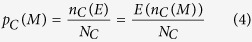


where *N*_*C*_ is the total number of patient in cancer type *C*, and *n*_*C*_(*M*) is the number of patient harboring a specific pfsSNV *M* in cancer type *C. n*_*C*_(*E*) is the expected number of patient in cancer type *C* for a given pfsSNV *M* for any functional types. Then the p-value was calculated as the sum of probabilities of observing number of patients the same as or more extreme (larger if the observed number of patients is larger than expected number, *E*(*n*_*C*_(*M*)), and lower if the observed number is smaller than *E*(*n*_*C*_(*M*))) than the observed number of patients *n*_*C*_(*O*) in the sample with the same cancer, *N*_*C*_.


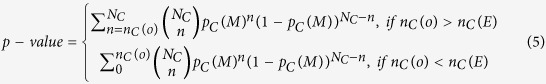


This approach takes into consideration the differences in cancer’s mutational rate and rank the pfsSNVs enriched within cancers despite the sparseness of somatic mutation among patients.

After the log transformation, p-values are visualized in Manhattan plot where horizontal axis represent chromosome from 1 to 23. The cutoff line was calculated as 2E-6 using Bonferroni approach. Lastly, we compared our prioritized pfsSNVs with a well-known cancer gene list: significantly mutated gene (SMG)^32^ and cancer gene census (CGC)^33^ to further annotate the key pfsSNVs list.

### Survival analysis

Identified key pfsSNVs were further investigated to see if any of them significantly affect patient survival. Patient clinical information was retrieved for TCGA samples from their FTP site (https://tcga-data.nci.nih.gov/tcgafiles/ftp_auth/distro_ftpusers/anonymous/tumor/). For each key pfsSNV in a specific cancer type that we identified through the prioritization process, based on the presence of the given pfsSNV, patients were divided into two groups with clinical factors that may affect patient survival. A log-rank test was applied to test the death time distributions between two groups. Then the Cox model was used to adjust factors like age at initial diagnosis, pathological stage and gender. SAS 9.3 was used to perform this analysis.

## Results and Discussion

### Impact of SNVs on protein functional sites

In this study, we expanded the scope of our previous study^8^ for better evaluation of mutational profile among various protein PTMs, active and binding sites. Tables 1 and 2 summarizes our data collection for both the current study and our previous study^8^. Table 1 shows total number of germline mutation, somatic mutation, and protein functional sites collected in both previous and current datasets. In Table 2, somatic and germline pfsSNV from Table 1 are split into major protein functional site types and summarized. The number of somatic mutations increased from 994,339 to 1,840,711 (1,272,878 non-synonymous, 476,087 synonymous and 91,746 stop codon). The number of germline mutations increased from 710,946 to 1,501,666 (937,634 non-synonymous, 541,029 synonymous and 23,003 stop codons). The number of protein functional sites increased from 259,216 to 268,478. After mapping both somatic and germline variations to protein functional site dataset, the number of pfsSNVs increases from 38,549 to 51,138 (31,999 somatic and 19,139 germline). We divided our pfsSNVs into four groups: non-synonymous germline mutation (non-SG), non-synonymous somatic mutation (non-SS), synonymous germline mutation (SG) and synonymous somatic mutation (SS) because each one of these mutation type has its own biological meaning, and therefore should be analyzed separately. Additionally, we enlarged the testable SNV dataset by incorporating predicted gain of N-linked glycosylation and phosphorylation site. It is common that SNV caused gain of PTM sites to be ignored in many HTS based proteome-wide analysis until recently^34^,^35^,^36^. We found a total number of 344,239 SNVs that cause gain of phosphorylation sites across 18,259 proteins and 17,921 SNVs that cause gain of N-linked glycosylation sites across 8,354 proteins.

**Figure 2:**
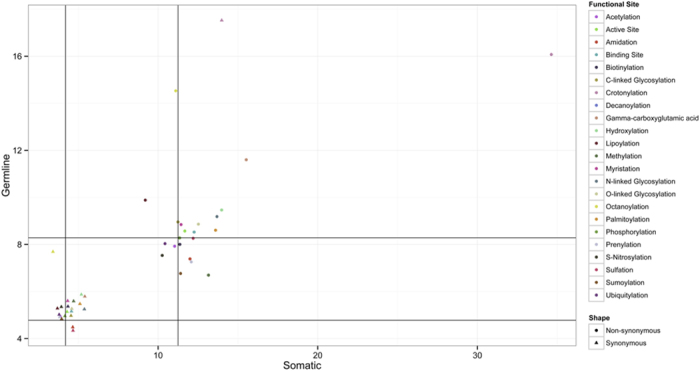
Synonymous and non-synonymous SNV occurrence ratio among different types of protein functional site. The values on each axis show, for each PTM type, the percentage of its site occupied by SNVs. X-axis shows the somatic mutation percentage and Y-axis shows germline mutation percentage. Dot and triangle markings represent non-synonymous and synonymous mutations respectively. Each protein functional site type was shown in different color as per the legend. Linear lines in the figure show global ratio for each mutation type.

In Fig. 2, for each protein functional site type, we calculated the percentage of its site impacted by somatic and germline SNVs (See Supplementary Table 1). In the scatter plot, X-axis and Y-axis indicate somatic and germline mutation percentages respectively while the dot and triangle represents non-synonymous and synonymous variation percentages respectively. Linear reference lines in the matrix show the global expected percentages. We can see from Fig. 2, for germline mutations, synonymous (lower reference line on Y axis) and non-synonymous (upper reference line on Y axis) SNVs cluster near the average reference lines. For somatic variations, synonymous and non-synonymous mutations also cluster near the averages (left reference line on X axis for synonymous; right reference line on X axis for non-synonymous). We can see that pfsSNV occurrence is around the global average percentages except for crotonylation sites, for which there are much more germline and somatic SNVs than the average. Outliers on the plot could be caused due to small sample size, for instance, crotonylation sites has higher synonymous and non-synonymous germline mutation occurrence than reference line but this is calculated based on just 79 data points.

**Figure 3:**
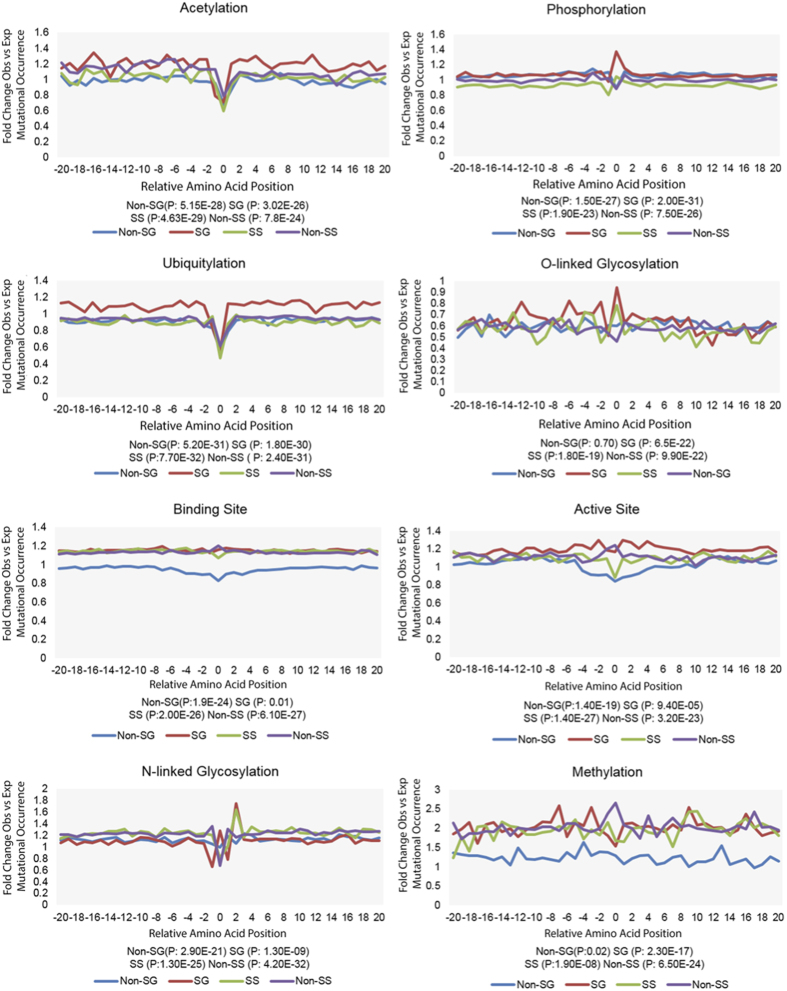
Occurrence ratio of SNV on the protein functional site neighboring region. Occurrence ratio of synonymous somatic (SS), synonymous germline (SG), non-synonymous somatic (non-SS) and non-synonymous germline (non-SG) mutations +/−20 amino acid from protein functional sites. Y-axis shows fold of change of SNV occurrence on corresponding amino acid position. Different SNV types are represented as different colors. Value 0 on X-axis indicates the PTM site. T and P represent one sample *t*-test of the PTM site comparing with its neighboring. P represents the p-value of corresponding one sample *t*-test.

Instead of just focusing on the exact protein functional sites (such as PTM and active/binding sites) we also evaluated the preponderance of SNVs upstream and downstream of the functional site. Figure 3, plots all the SNV occurrence of residues with +/−20 amino acids around the functional site (see Supplementary Table 3a,b,c and d for plots of all 25 types). In most of the PTM sites, non-synonymous germline mutation (non-SG) shows either relatively low occurrence or similar rates when compared to neighboring regions. This result is consistent with the high evolutionary conservation of functional sites^15^,^37^. On the other hand, synonymous germline mutation shows mixed occurrence across different PTMs types with lower than expected occurrences for in some of the sites. It is interesting to note that several studies have shown that synonymous mutation can affect protein function^16^,^38^,^39^.

Out of 8,357 experimental confirmed acetylation sites in the human proteome, 691 lose acetylation site due to somatic mutation and 432 lose acetylation site due to germline mutations. In 22,524 ubiquitination sites in the human proteome, 1,562 ubiquitination sites are lost due to somatic mutations and 1052 ubiquitination sites are lost due to germline mutations. In comparison with our previous paper, the number of loss of acetylation sites and ubiquitination sites increased by 48 and 559 respectively. Dysregulation of both acetylation and ubiquitination processes may cause cancer initiation and it has been observed by others that there are frequent mutations in acetylation and ubiquitination sites which potentially can drive cancer^40^,^41^,^42^. For acetylation, different modified sites have distinct regulatory effects, even in the same protein (e.g. malate dehydrogenase 2)^41^. In another study, researchers found that both acetylation and deacetylation of p53 on different amino acids could either promote or block tumorigenesis^43^. Its complexity leads to the disunity of acetylation function in cancers. Our analysis shows low non-synonymous somatic (non-SS) mutation occurrence on acetylation sites suggesting that in cancer these sites are still less prone to mutations. In terms of ubiquitination, it can be seen that ubiquitination sites are less tolerant to SNVs (relatively conserved) compared with its neighboring region.

In our current dataset, we identified 7,373 somatic mutations and 5,282 germline mutations that cause loss of phosphorylation sites. Previous studies found high enrichment of mutations causing gain or loss of phosphorylation sites and they may be considered as key features in cancer occurrence^34^. High activity of kinases is essential to maintain the tumor malignant phenotype (oncogene addiction)^44^. It is consistent with our result that non-synonymous mutations (non-SS) show low occurrence at phosphorylation sites. It is also possible that the low occurrence on phosphorylation site may be caused by the relatively small number of cancer related genes^45^.

We identified 2,084 somatic mutations and 1,040 germline mutations that can cause loss of enzyme active site. In Fig. 3, the non-synonymous somatic mutation occurrence at active site is relatively higher than that at its surrounding regions. However, when the enzyme active site is considered, its role in cancer is also dependent on the feature of the protein (oncogene or tumor suppressor gene). For example, in breast cancer, overexpression of BCRP (breast cancer resistance protein) with its intact active site could cause drug resistance, while mutation in the active site of α-fetoprotein (AFP) could reduce breast cancer risk^46^. These mutations can impact enzymes to metabolize different substrates^47^, leading to pathological processes. In Fig. 3, the non-synonymous somatic mutation occurrence at active site is relatively higher than at its surrounding regions. Synonymous somatic mutation, on the other hand, has a low occurrence rate at active sites. This bias may be caused by the highly structure-dependent catalytic activity (stable structure is crucial for function)^48^. At ligand binding sites, 16,630 somatic mutations and 25,074 germline mutations were identified. For binding sites, studies have found their relationship with disease occurrence in terms of mutations^49^,^50^. Binding site analysis shows little SNV occurrence difference compared to its neighboring regions for SG, SS and non-SS, but overall low mutation occurrence in the entire functional region for non-SG. However, we would like to mention that binding sites can contain multiple sites which are not sequentially placed in the sequence. Our analysis focuses on short regions (see materials and methods), and counting each residue as one binding sites and the immediate region around it thus providing a practical and comparable evaluation of binding sites and other protein functional sites.

For methylation sites, we identified 208 somatic mutations and 74 germline mutations. It is interesting to note that the overall occurrence of SG, SS and non-SS is as high as two fold compared to the background occurrence. In particular, the non-SS mutation occurrence at the methylation sites is relatively higher than other mutation type and also their surrounding regions. Methylation regulates transcription factor binding affinity, and therefore, controls the expression level of the downstream target genes^51^. In consideration of cancer development, previous study suggests lysine-to-methionine substitution at methylation sites could cause loss of methylation and function in a variety of pathologies. And in our results, the relatively high non-SS mutation occurrence of methylation may suggest its primary role in either promoting oncogenes or suppressing tumor suppressor genes.

3,217 somatic mutations and 2,630 germline mutations were identified on N-linked glycosylation sites. Figure 3 also shows that the SNV occurrence at the N-linked glycosylation site and its surrounding amino acids (−1, +1 and +2) are much higher than others. Non-synonymous somatic mutation shows a deep dip at the N-linked glycosylation (0.67). In our previous study^52^, we found slightly lower frequency of all kinds of missense mutations in N (position 0) than the non-glycosylated motifs. This is also consistent with the higher conservation of glycoslylated asparagines as compared with the non-glycosylated ones^53^. Such a low mutation occurrence in the cancer genome implies its contribution and its role in cancer. In addition, somatic synonymous mutations (0.89) also show a similar trend at N-linked glycosylation sites. This also suggests that it is important to maintain N-linked glycosylation site undisrupted. Although, it is quite possible the overall functional impact is maintained through the heterogeneity of the glycans at the sites in normal vs. cancer tissues^54^.

The NX(S/T) amino acid sequon (asparagine for N, any amino acid except proline for X, and either serine or threonine for S/T) is considered as a requirement for N-glycosylation^52^. This could explain the low occurrence of the two types of synonymous mutations (germline and somatic) at the amino acid of position +1 (X) but higher rates for non-synonymous mutation, and high rate (SS: 1.64, SG: 1.75) at position +2 (alternation of serine and threonine, S/T). Additionally, we found that the amino acid at ‘−1’ position also has lower synonymous germline mutation occurrence, which suggests possible effects of “silent” mutations at this site.

In terms of O-linked glycosylation, 126 somatic mutations and 115 germline mutations were identified impacting the PTM site. O-linked glycosylation is known to be important in bearing tumor associated antigens and also involved in several physiological and pathological processes^55^,^56^,^57^. One interesting finding is that O-linked glycosylation sites is the only functional site type showing overall low occurrences across the entire functional site region in terms of all mutation types (non-SG: 0.60, SG: 0.64, SS: 0.57, non-SS: 0.59).

### Pan-cancer view of somatic mutation occurrence on protein functional sites

**Figure 4:**
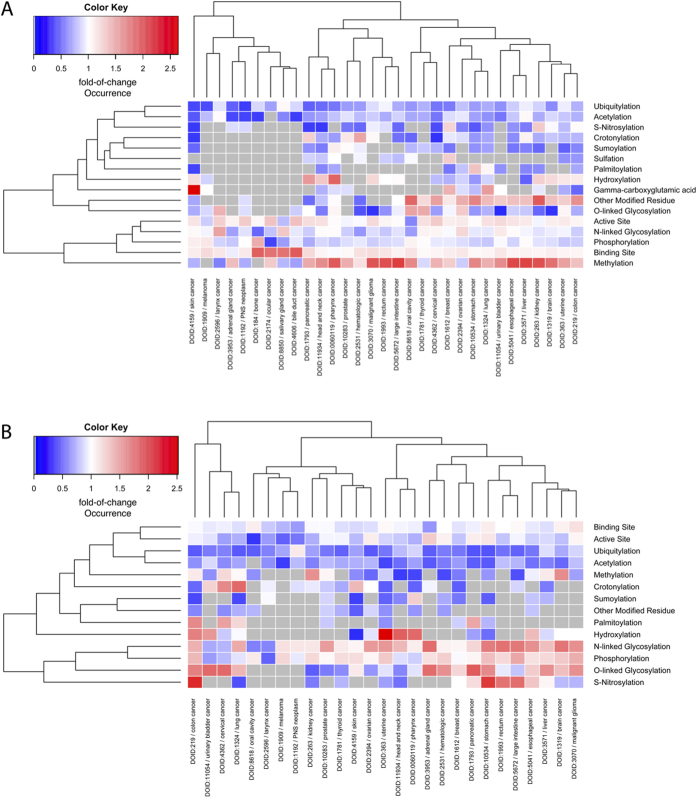
Pan-caner hierarchical clustering of non-synonymous (**A**) and synonymous (**B**) somatic mutation occurrence on protein functional site region. Figure shows cancer SNV occurrence at PTM site vs somatic SNV occurrence at a neighboring region for different cancer types. Color indicates fold of change of somatic SNV occurrence. Red color indicates overrepresentation while blue indicates under-representation. Grey color means that there is no detected somatic SNV on corresponding PTM type for corresponding cancer.

For pan-cancer analysis, cancer Disease Ontology (DO) slim^23^ was used to unify the cancer types. The observed and expected somatic mutation occurrence on each functional type was then calculated. Figure 4 shows the pan-cancer heatmap of somatic mutation occurrences across functional sites (Fig. 4A: non-synonymous, Fig. 4B: synonymous). The mutation occurrence is indicated by ratio of change compared to the cancer type specific global ratio. Color in the figure indicates either the over-representation (red) or under-representation (blue) of pfsSNVs while white indicates no SNV occurrence difference between functional sites and neighboring sites. The grey color indicates the absence of pfsSNVs for the corresponding cancer type. Our assumption is that, since functional sites are generally conserved, the high/low ratio of somatic pfsSNVs occurrence on these sites implies the loss/gain of function for them and their possible roles in tumorigenesis.

The pan-cancer view of observed/expected SNVs shown in Fig. 4A displays unique patterns of nsSNV occurrence on functional sites (compared to neighboring site) in different cancer types. The variation occurrence at ubiquitination and acetylation sites is lower (blue color) at these PTM sites across almost all cancer types. On the other hand, the methylation site shows higher nsSNV occurrence (red color) in PTM site for majority of the cancer types. Active sites, binding sites, phosphorylation sites, and N-linked glycosylation sites show insignificant fold-change between PTM sites and neighboring sites. Similarly, for synonymous mutations (Fig. 4B), ubiquitination and acetylation site show an overall low somatic synonymous mutation occurrence at PTM sites across almost all the cancer types. However, unlike in non-synonymous mutation, methylation sites show mixed mutation occurrence across cancer types. Phosphorylation sites and N-linked glycosylation shows an increased synonymous mutation occurrence in multiple cancer types.

#### Identification of key pfsSNVs across multiple cancer types

**Figure 5:**
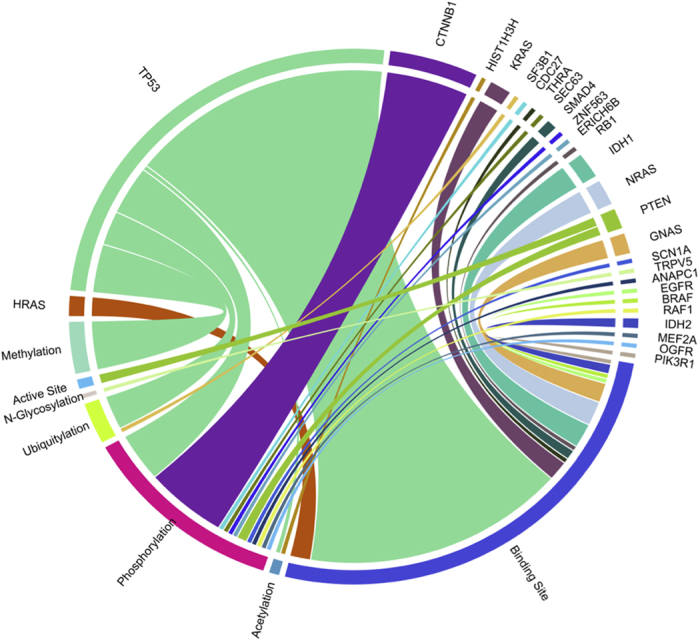
Circos plot of gene level summarization of 142 key pfsSNVs across five and more cancer types. Bands are colored by genes, and connect between gene and various types of protein functional sites. Note that, in 142 key pfsSNVs, all key pfsSNVs on CTNNB1 occur on phosphorylation site and all key pfsSNVs on RAS subfamily occur on binding site.

Out of the 31,999 germline pfsSNVs and 19.139 somatic pfsSNVs, we found that 142 pfsSNVs exist across more than five cancer types, which we considered as key pfsSNVs (see Supplementary Table 6 for pfsSNVs in more than 3 cancer types). Table 3 displays the top 20 pfsSNVs with respect to number of associated cancer types. In addition, Fig. 5 shows their SNV-functional site relationship in the Circos plot^58^. From both Table 3 and Fig. 5 we can see that TP53, one of the most well-known oncogenes, with 79 out of 142 key pfsSNVs on that protein. We also want to emphasize pfsSNVs that exist on genes other than TP53. Since TP53 is a well-known oncogene, we emphasize top 20 pfsSNVs associated with multiple cancer types with TP53 excluded in Table 4: NRAS, CTNNB1, NRAS, GNAS, KRAS, HRAS and PTEN. It is clear that some genes harbor more key pfsSNVs than others as shown in Fig. 5. 14 out of 142 key pfsSNVs, including two of the top 20 pfsSNVs are found within CTNNB1 which is an important component of the canonical Wnt signaling pathway. It is interesting to note that all these key pfsSNVs are affecting protein phosphorylation sites between position 29 to 45. This finding confirms previous studies’^59^ claims that SNVs and overexpression of CTNNB1 are associated with many cancers: a large number of SNVs cluster on the N-terminal segment of CTNNB1, the β-TrCP binding motif.

Other than TP53 and CTNNB1, many key members of Ras subfamily, such as NRAS, GNAS, KRAS and HRAS harbor SNVs across multiple cancer types. Figure 5 shows that virtually all the pfsSNVs on Ras subfamily are located on binding site. However, multiple alignment of NRAS, GNAS, KRAS and HRAS shows that most of the key pfsSNVs within these four genes occurs at the same position (RASN_HUMAN Q61), a well-known position responsible GAP-mediated GTP hydrolysis. SNVs on this residue disturb Ras signaling control and eventually trigger tumorigenesis by activate genes involved in cell growth, differentiation and survival^60^.

#### Identification of key pfsSNVs that are enriched in patients with specific cancer types

**Figure 6:**
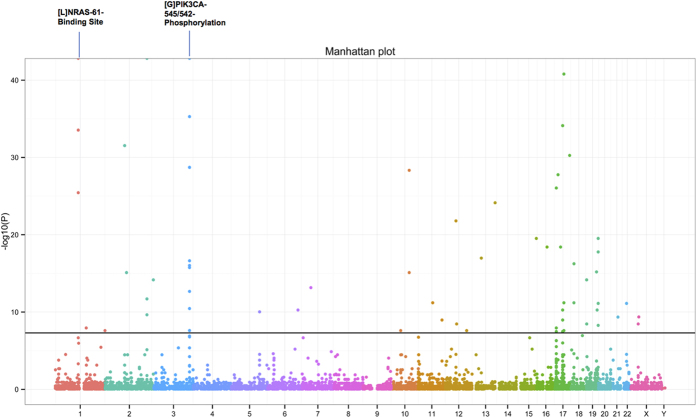
Manhattan plot of pfsSNVs enriched in patients with specific cancer types. X-axis indicates chromosome from 1 to 23 and X, Y in different colors. Each dot in the figure represents a pfsSNV with –log10 (p-value) calculated from a binomial test. Cutoff was set as -log10 (5e-8). A total number of 77 pfsSNVs are statistically significant in specific cancer type. [L] and [G] indicate loss of PTM/active/binding site and gain of PTM/active/binding site respectively. As marked in the figure, [L]NRAS-61-Binding Site and [G]PIK3CA-545/542-Phosphorylation significantly associate with multiple cancer type.

To ensure we do not miss any pfsSNVs that occur repetitively among patients within a specific cancer type, we performed Binomial test using a dataset combining known and predicted gaining/losing pfsSNV sites. This dataset includes 19,337 loss of functional site causing pfsSNVs, 10,991 gain of N-glycosylation sites, and 208,507 gain of phosphorylation sites. Log p-values for each pfsSNVs were used for visualization in Fig. 6 (See Supplementary Table 5 for all pfsSNVs with p-value). Based on our threshold (p-value = 2E-6 using the Bonferroni adjustment), a total number of 77 pfsSNVs (57 gain of phosphorylation site pfsSNVs, 3 gain of glycosylation site pfsSNVs, 12 loss of binding site pfsSNVs, 3 loss of phosphorylation site and 2 loss of active sites) were identified to be significant in specific cancer types. Table 5 shows the top 20 pfsSNVs with significant p-value associated with specific cancer types. [L] and [G] indicate loss of functional site and gain of functional site, respectively. Supplementary Table 5 shows p-values for all 24,668 pfsSNVs associated with specific cancer type. For example, the gain of phosphorylation site pfsSNV PIK3CA-545-E-K is significantly associated with as many as six cancer types (63 patients in breast cancer, 28 patients in head and neck cancer, 33 patients in cervical cancer, 19 patients in colon cancer, 14 patients in uterine cancer, 11 patients in stomach cancer).

Pan-cancer analysis mentioned above identified a total number of 210 key pfsSNVs, among which 142 exist across more than five cancer types and 77 pfsSNVs are significantly enriched in patients with specific cancer type. All these 210 key pfsSNVs belong to 60 genes. For the purpose of comparison with key cancer genes found in other studies, we retrieved the significantly mutated gene (SMG) set found by MutSig suite^32^ and cancer gene census (CGC) from COSMIC^33^. By mapping SMG (260 genes), CGC (573 genes) and key pfsSNVs (60 genes), we found our key pfsSNVs map to 18 and 20 genes from SMG and CGC respectively. Moreover, we found 17 of them exist in all three datasets. Table 6 shows the list of these 17 genes with 132 pfsSNVs within them. These 17 genes and their key pfsSNVs which are 1) present in the list of 260 SMG set, 2) present in the list of 573 CGC gene set, 3) have key pfsSNVs which either exist across multiple cancer types or are significantly associated with specific cancer type.

#### Existing knowledge on 132 key pfsSNVs

Many of these 132 pfsSNVs and their genes have been described in the previous studies. One study showed BRAF is commonly activated by somatic point mutation in human cancer, and may suggest therapeutic potentials particularly in malignant melanoma^61^. The BRAF L597R missense mutation, which falls in the protein’s kinase domain, has been reported in primary ovarian cancer (OV)^61^ and lung adenocarcinoma (LUAD), and may become a chemotherapy target for a subset of LUAD patients. CTNNB1 mutation are found in the GSK3-beta phosphorylation sites, such as S37, T41, and such mutations have been implicated in ovarian tumorigenesis^62^. It has been also suggested that CTNNB1 has a higher rate of phosphorylation-related mutations in skin cancer and performs a critical role in hair matrix cell cancer development^63^. The EGFR T790M alteration is called the “gatekeeper” mutation, which is frequently described in lung cancers; it mediates resistance to maximally tolerated dosing of HKI-272 as well as EGFR kinase inhibitors (gefitinib and erlotinib) in about half of cases^64^,^65^,^66^. One study also indicated that this drug resistance mutation may also be linked with lung cancer genetic susceptibility^67^. HRAS shows high incidence of activating mutations at Q61 in drug-induced skin cancer^68^,^69^,^70^. As for KRAS mutations at Q61 in lung cancer, Q61R is observed in a great portion of urethane-induced tumors from wild-type mice, however, Q61L appears in the majority of tumors from KRAS heterozygous mice. KRAS Q61 mutations may also play an important role in melanoma photocarcinogenesis^71^. NRAS Q61 is predominant in malignant melanoma, being a potential therapeutic target in this cancer^72^. Mutated NRAS at Q61 also shares similarities in signaling among various cancer types, and inhibition of both the MAPK and PI3K/AKT/mTOR pathways reduces cell viability in all cancers harboring this mutation^73^. IDH1 mutation at R132 is demonstrated to be tissue-specific, and may play a special role in high-grade gliomas with prognostic value for survival^74^,^75^. IDH1 and IDH2 mutations are common in AML (acute myeloid leukemia), and are associated with the accumulation of metabolite 2-hydroxyglutarate, which is affected by neomorphic enzyme activity^76^,^77^,^78^.

### Identification of key pfsSNVs that affect patient survival

Although there are many studies that attempt to connect mutations to patient survival there are very few attempts to connect loss or gain of protein function to mortality. Identifying SNVs that directly lead to gain or loss of functional sites can help biologists focus on specific biochemical processes that might be impacted due to the variation. The amount of clinical data available from TCGA is limited because of the length of the study and the type of information that has been collected so far. Nonetheless, it is possible to showcase how one can filter SNVs for further evaluation which can assist in translating genomics efforts to actionable therapeutics and diagnostics. Below is an example of how such analysis can be performed.

**Figure 7:**
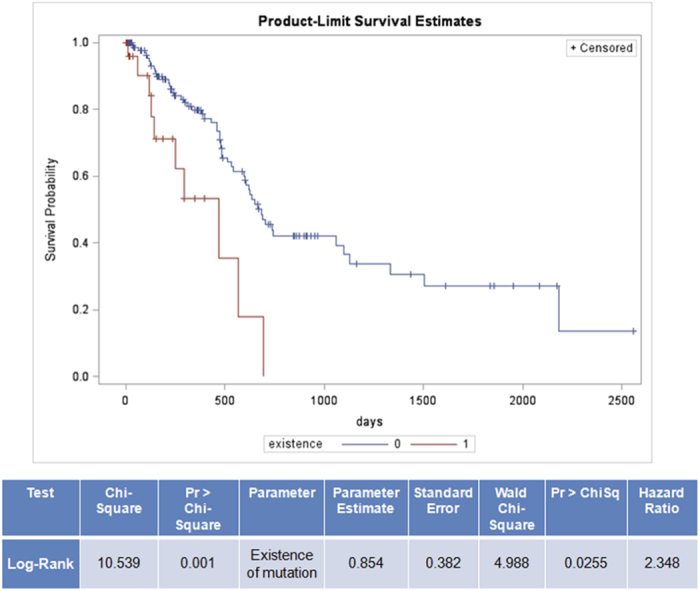
Kaplan-Meier plot of pancreatic cancer patient survival based on the existence of MEF2A-105-Y-C-Phosphorylation. X-axis indicate days of survival and Y-axis indicates survival probability. Red and blue lines indicate survival time of pancreatic cancer patients with and without such mutation respectively. Log-rank test shows that, comparing with patients with MEF2A-105-Y-C-Phosphorylation, patients without this pfsSNV survive significantly longer with adjusted p-value of 0.0255. The hazard ratio is 2.348.

We started from the pool of above identified 77 key pfsSNVs that are significantly enriched in a specific cancer type after adjusting multiple testing. Then we grouped patients based on ‘having’ or ‘not having’ a pfsSNV. From Cox regressions adjusting for age at initial pathological diagnosis, gender and clinical stage 27 out of the 77 mutations increase the risk of cancer (Hazard Ratio >1). Most of the candidate pfsSNVs occur in very small number of patients, and sometimes no death case has been observed in the small number of patients with the candidate pftSNVs, in which cases data cannot provide a valid hazard ratio estimate. We identified 3 pfsSNVs causing significant higher or lower mortality risks (See Fig. 7, Supplementary Figures 1 and 2). One example found in pancreatic cancer patients with and without the MEF2A-Y105C-Phosphorylation site was found to have statistically significant different mortality risk (p-value = 0.0012) from a log-rank test. Even after adjusting for age at initial diagnosis, pathological stage and gender using a Cox model, the mortality risks in the presence of MEF2A-Y105C is 2.348 times higher than those without MEF2A -Y105C (adjusted p-value = 0.0255). Figure 7 shows that the Kaplan-Meier estimates in the survival probabilities over days since diagnosis of the two groups are well separated. MEF2A is a transcriptional factor which binds to MEF2 element and activates numerous growth factors. It plays diverse roles in the control of cell growth, survival and apoptosis^79^,^80^,^81^. Although many studies have been performed on MEF2’s role in muscle and neuron, its role in pancreatic cancer remains unclear.

Although we found few pfsSNVs which appear to be associated with survival, we would like to point out the limitation of the survival analysis as our sample size is quite small among ‘having mutation’ group and ‘no mutation’ groups. When no death has been observed in the small group of participants with a specific mutation, the impact of the pftSNV on mortality are not estimable. Because no studies can enroll an infinite sample size or follow participants indefinitely, this analysis has no intention to overcome this inherent limitation of data, while just as we stated in the beginning of this section, it serves a showcase of potential impact of pfsSNVs to evoke more interest for follow-up studies.

## Conclusion

We have comprehensively investigated the interplay between protein functional sites and SNVs. Each type of protein functional site shows a distinct SNV frequency in synonymous somatic mutations, synonymous germline mutations, non-synonymous somatic mutations, and non-synonymous germline mutations. Our experiments show that, at least for the majority of protein functional site types, non-synonymous germline mutations occur less frequently. We believe that these sites are, as expected, more evolutionarily conserved because of their functionality. Except for acetylation and ubiquitination sites, other protein functional site types show diverse variation frequencies between synonymous germline SNVs, synonymous somatic SNVs and non-synonymous somatic SNVs. Investigation of whether the protein functional sites of tumor tissue tends to accumulate or reject SNVs at functional sites provides insights on the effect of SNVs impacting each type of protein functional sites. For synonymous variations, although previous studies show that such variations can affect protein function by changing expression level, current understanding of the effects of synonymous mutations is still limited. However, some protein functional site types show significant synonymous variation frequency changes, for example, O-linked glycosylation site contains significantly high frequency of both germline (*t* statistic = −19.35, p-value = 6.5E-22) and somatic mutation (*t* statistic = −16.54, p-value = 1.80E-19).

Although a number of studies have been conducted to discover significant mutated genes in cancer^82^,^83^,^84^, our study takes steps forward by targeting the impact of key SNVs on amino acids which can be further evaluated through wet-laboratory experimentations. The top pfsSNVs exist among well-known oncogenes, such as DNA binding sites and zinc binding sites within TP53, and GEF interaction sites within NRAS. This study identified several highly mutated regions such as position 29–45 in CTNNB1. To make the key pfsSNVs comprehensive, we conducted a binomial test using both loss and gain of functional site causing pfsSNVs. This approach identified 77 pfsSNVs enriched in patients with specific cancer types which are good candidates for further investigation in terms of their biological function and effect in tumor growth. The identification of key pfsSNVs has its value not only in facilitating the investigation of tumorigenesis mechanism, but also in evaluating the risk of developing cancer. Identified pfsSNVs can be further evaluated using resources such as MutationAligner^85^,^86^, and other mutation analysis services^87^,^88^,^89^.

## Additional Information

**How to cite this article:** Pan, Y. *et al*. Distribution bias analysis of germline and somatic single-nucleotide variations that impact protein functional site and neighboring amino acids. *Sci. Rep.* **7**, 42169; doi: 10.1038/srep42169 (2017).

**Publisher's note:** Springer Nature remains neutral with regard to jurisdictional claims in published maps and institutional affiliations.

## Supplementary Material

Supplementary Figures

Supplementary Table 1

Supplementary Table 2

Supplementary Table 3

Supplementary Table 4

Supplementary Table 5

Supplementary Table 6

## Figures and Tables

**Table 1:** Position based* summary of comparison between the previous and current datasets.

	Somatic Mutation	Germline Mutation	Functional Site	Somatic pfsSNV Mapped	Germline pfsSNV Mapped	Total Mutations Mapped
**Previous Dataset**	994,339	710,946	259,216	25,390	13,159	38,549
**Current Dataset**	1,840,711	1,501,666	268,478	30,848	18,619	49,467

^*^Statistics summarized in Table 1 is amino acid position based where different functional types occupying the amino acid position are counted as one.

**Table 2:** pfsSNVs based* summary of the previous and current datasets.

	Previous Version of Dataset			Current Version of Dataset			%Increase
	Somatic Mutation	Germline Mutation	Total	Somatic Mutation	Germline Mutation	Total	Increases by
**Acetylation**	512	351	863	691	432	1,123	30.1%
**Ubiquitination**	1,214	841	2055	1562	1052	2,614	27.2%
**Phosphorylation**	5,466	3917	9383	7373	5282	12,655	34.9%
**N-linked glycosylation sites**	2,375	1,997	4,372	3,217	2,630	5,847	33.7%
**O-linked glycosylation**	97	108	205	126	115	241	17.6%
**Methylation**	163	61	224	208	74	282	25.9%
**Crotonylation**	42	10	52	57	22	79	52.0%
**Nitrosylation**	32	43	75	51	48	99	32.0%
**Active sites**	1,574	811	2385	2,084	1,040	3,124	31.0%
**Binding sites**	12,286	6,395	18,681	16,630	8,444	25,074	34.2%
**Total**	23,761	14,534	38,295	31,999	19,139	51,138	33.5%

^*^Statistics summarized in Table 2 is pfsSNVs based where different functional types occupying the amino acid position are counted separately.

**Table 3:** Top 20 pfsSNVs^1^ based on the number of associated cancer type count.

Gene Name	UnProtKB AC	Variation	Functional Site	Cancer Type Count
TP53	P04637	R273C	Binding Site	31
TP53	P04637	R248Q	Binding Site	28
TP53	P04637	R248W	Binding Site	28
TP53	P04637	R273H	Binding Site	27
TP53	P04637	H179Y	Binding Site	26
NRAS	P01111	Q61K	Binding Site	24
TP53	P04637	C176F	Binding Site	23
TP53	P04637	C275Y	Binding Site	22
NRAS	P01111	Q61R	Binding Site	21
CTNNB1	P35222	T41A	Phosphorylation	21
TP53	P04637	C176Y	Binding Site	20
TP53	P04637	H179R	Binding Site	20
TP53	P04637	K132N	Ubiquitylation	19
TP53	P04637	C238F	Binding Site	19
TP53	P04637	C242F	Binding Site	19
TP53	P04637	R248L	Binding Site	19
TP53	P04637	S241F	Binding Site	18
TP53	P04637	C242Y	Binding Site	18
CTNNB1	P35222	S33C	Phosphorylation	18
TP53	P04637	C238Y	Binding Site	17

^1^pfsSNV: Protein functional site affecting SNV.

**Table 4:** Top 20 pfsSNVs based on the number of associated cancer type count (TP53 excluded).

Gene Name	UnprotKB ID	Variation	Functional Site	Cancer Type Count
NRAS	P01111	Q61K	Binding Site	24
CTNNB1	P35222	T41A	Phosphorylation	21
NRAS	P01111	Q61R	Binding Site	21
CTNNB1	P35222	S33C	Phosphorylation	18
GNAS	P63092	R201C	Binding Site	16
GNAS	Q5JWF2	R844C	Binding Site	16
KRAS	P01116	Q61H	Binding Site	16
HRAS	P01112	Q61L	Binding Site	15
NRAS	P01111	Q61L	Binding Site	15
PTEN	P60484	R130Q	Active Site	15
CTNNB1	P35222	S33F	Phosphorylation	14
CTNNB1	P35222	S37C	Phosphorylation	14
CTNNB1	P35222	S37F	Phosphorylation	14
CTNNB1	P35222	S45F	Phosphorylation	14
GNAS	P63092	R201H	Binding Site	14
GNAS	Q5JWF2	R844H	Binding Site	14
CTNNB1	P35222	T41I	Phosphorylation	13
CTNNB1	P35222	S45P	Phosphorylation	13
KRAS	P01116	Q61K	Binding Site	13
KRAS	P01116	Q61L	Binding Site	13

**Table 5:** Top 20 pfsSNVs enriched in patients with specific cancer type (full list in Supplementary Table 5).

PfsSNVs	Cancer Type	PfsSNVs Associated Sample	Total Sample	P-Value
[G]^2^PIK3CA-E545K-Phosphorylation	DOID:1612/breast cancer	63	973	9.62E-85
[L]NRAS-Q61R-Binding Site	DOID:4159/skin cancer	44	370	9.94E-51
[G]PIK3CA-E542K-Phosphorylation	DOID:1612/breast cancer	41	973	4.43E-48
[G]CDC27-A274D-Phosphorylation	DOID:1793/pancreatic cancer	44	210	1.59E-41
[G]PIK3CA-E545K-Phosphorylation	DOID:4362/cervical cancer	33	198	5.25E-36
[G]KRTAP4-L161V-Phosphorylation	DOID:1793/pancreatic cancer	39	210	7.70E-35
[L]NRAS-Q61K-Binding Site	DOID:4159/skin cancer	33	370	2.91E-34
[G]ANKRD36-T998S-Phosphorylation	DOID:1793/pancreatic cancer	37	210	2.94E-32
[G]EVPL-R336S-Phosphorylation	DOID:1793/pancreatic cancer	36	210	5.47E-31
[G]PIK3CA-E545K-Phosphorylation	DOID:11934/head and neck cancer	28	508	1.92E-29
[L]PTEN-R130G-Active Site	DOID:363/uterine cancer	28	305	4.59E-29
[G]NCOR1-Y20S-Phosphorylation	DOID:1793/pancreatic cancer	34	210	1.72E-28
[L]TP53-R273C-Binding Site	DOID:1319/brain cancer	31	287	8.97E-27
[L]NRAS-Q61R-Binding Site	DOID:1781/thyroid cancer	27	390	3.68E-26
[G]UPF3A-V70L-Phosphorylation	DOID:1793/pancreatic cancer	31	210	7.35E-25
[G]KRT8-R23C-Phosphorylation	DOID:1793/pancreatic cancer	29	210	1.61E-22
[L]MEF2A-Y105C-Phosphorylation	DOID:1793/pancreatic cancer	27	210	3.02E-20
[G]ZNF814-A337V-Phosphorylation	DOID:1793/pancreatic cancer	27	210	3.02E-20
[G]SALL1-S159G-Phosphorylation	DOID:1793/pancreatic cancer	26	210	3.88E-19

[L]^1^: Loss of protein functional site.

[G]^2^: Gain of protein functional site.

**Table 6:** 132 pfsSNVs that satisfy key pfsSNVs identification criteria.

Gene Name	UniProtAC	Variation	Functional site	Gene Name	UniProtAC	Variation	Functional site
BCOR	Q6W2J9	N1459S	[G]Phosphorylation	TP53	P04637	N239D	[L]Binding Site
BRAF	P15056	L597R	[L]Binding Site	TP53	P04637	C242S	[L]Binding Site
CTNNB1	P35222	T41A	[L]Phosphorylation	TP53	P04637	R273L	[L]Binding Site
CTNNB1	P35222	S33C	[L]Phosphorylation	TP53	P04637	R280T	[L]Binding Site
CTNNB1	P35222	S33F	[L]Phosphorylation	TP53	P04637	N239S	[L]Binding Site
CTNNB1	P35222	S37C	[L]Phosphorylation	TP53	P04637	C275F	[L]Binding Site
CTNNB1	P35222	S37F	[L]Phosphorylation	TP53	P04637	C176S	[L]Binding Site
CTNNB1	P35222	S45F	[L]Phosphorylation	TP53	P04637	H179L	[L]Binding Site
CTNNB1	P35222	T41I	[L]Phosphorylation	TP53	P04637	R273S	[L]Binding Site
CTNNB1	P35222	S45P	[L]Phosphorylation	TP53	P04637	C238S	[L]Binding Site
CTNNB1	P35222	S33Y	[L]Phosphorylation	TP53	P04637	R273P	[L]Binding Site
CTNNB1	P35222	S37Y	[L]Phosphorylation	TP53	P04637	A276P	[L]Binding Site
CTNNB1	P35222	S33P	[L]Phosphorylation	TP53	P04637	R280G	[L]Binding Site
CTNNB1	P35222	S37A	[L]Phosphorylation	TP53	P04637	C238R	[L]Binding Site
CTNNB1	P35222	S37P	[L]Phosphorylation	TP53	P04637	S241Y	[L]Binding Site
CTNNB1	P35222	S33A	[L]Phosphorylation	TP53	P04637	R280S	[L]Binding Site
CTNNB1	P35222	S45C	[L]Phosphorylation	TP53	P04637	H179Q	[L]Binding Site
CTNNB1	P35222	S45Y	[L]Phosphorylation	TP53	P04637	S241C	[L]Binding Site
EGFR	P00533	T790M	[L]Binding Site	TP53	P04637	C242R	[L]Binding Site
HRAS	P01112	Q61L	[L]Binding Site	TP53	P04637	C277F	[L]Binding Site
HRAS	P01112	Q61K	[L]Binding Site	TP53	P04637	H179D	[L]Binding Site
HRAS	P01112	Q61R	[L]Binding Site	TP53	P04637	H179N	[L]Binding Site
HRAS	P01112	Q61H	[L]Binding Site	TP53	P04637	R273G	[L]Binding Site
IDH1	O75874	R132H	[L]Binding Site	TP53	P04637	C277Y	[L]Binding Site
IDH1	O75874	R132C	[L]Binding Site	TP53	P04637	S241A	[L]Binding Site
IDH1	O75874	R132G	[L]Binding Site	TP53	P04637	C242W	[L]Binding Site
IDH1	O75874	R132L	[L]Binding Site	TP53	P04637	R248G	[L]Binding Site
IDH1	O75874	R132S	[L]Binding Site	TP53	P04637	R248P	[L]Binding Site
IDH2	P48735	R172K	[L]Binding Site	TP53	P04637	R280I	[L]Binding Site
IDH2	P48735	R172S	[L]Binding Site	TP53	P04637	C176R	[L]Binding Site
KRAS	P01116	Q61H	[L]Binding Site	TP53	P04637	C275W	[L]Binding Site
KRAS	P01116	Q61K	[L]Binding Site	TP53	P04637	C176W	[L]Binding Site
KRAS	P01116	Q61L	[L]Binding Site	TP53	P04637	C275R	[L]Binding Site
KRAS	P01116	Q61R	[L]Binding Site	TP53	P04637	A276D	[L]Binding Site
NCOR1	O75376	Y20S	[G]Phosphorylation	TP53	P04637	A276T	[L]Binding Site
NRAS	P01111	Q61R	[L]Binding Site	TP53	P04637	R337C	[L]Methylation
NRAS	P01111	Q61K	[L]Binding Site	TP53	P04637	R213L	[L]Methylation
NRAS	P01111	Q61L	[L]Binding Site	TP53	P04637	R110L	[L]Methylation
NRAS	P01111	Q61H	[L]Binding Site	TP53	P04637	R213Q	[L]Methylation
NRAS	P01111	Q61E	[L]Binding Site	TP53	P04637	R110P	[L]Methylation
PIK3CA	P42336	E545K	[G]Phosphorylation	TP53	P04637	R213P	[L]Methylation
PIK3CA	P42336	E542K	[G]Phosphorylation	TP53	P04637	R337L	[L]Methylation
PIK3CA	P42336	N345K	[G]Phosphorylation	TP53	P04637	R110C	[L]Methylation
PIK3R1	P27986	N564D	[L]Binding Site	TP53	P04637	R209K	[L]Methylation
PTEN	P60484	R130G	[L]Active Site	TP53	P04637	R337H	[L]Methylation
PTEN	P60484	R130Q	[L]Active Site	TP53	P04637	S215R	[L]Phosphorylation
PTEN	P60484	D92E	[L]Active Site	TP53	P04637	T155N	[L]Phosphorylation
PTEN	P60484	Y155C	[L]Phosphorylation	TP53	P04637	T155I	[L]Phosphorylation
PTEN	P60484	Y68H	[L]Phosphorylation	TP53	P04637	S215I	[L]Phosphorylation
RB1	P06400	R661W	[L]Binding Site	TP53	P04637	T211I	[L]Phosphorylation
SF3B1	O75533	K700E	[L]Ubiquitylation	TP53	P04637	T155P	[L]Phosphorylation
SMAD4	Q13485	R361H	[L]Binding Site	TP53	P04637	S215G	[L]Phosphorylation
SMAD4	Q13485	R361C	[L]Binding Site	TP53	P04637	S215N	[L]Phosphorylation
TP53	P04637	R273H	[L]Binding Site	TP53	P04637	T155A	[L]Phosphorylation
TP53	P04637	H179Y	[L]Binding Site	TP53	P04637	T284P	[L]Phosphorylation
TP53	P04637	C176F	[L]Binding Site	TP53	P04637	K132N	[L]Ubiquitylation
TP53	P04637	C275Y	[L]Binding Site	TP53	P04637	K132R	[L]Ubiquitylation
TP53	P04637	C176Y	[L]Binding Site	TP53	P04637	K132E	[L]Ubiquitylation
TP53	P04637	H179R	[L]Binding Site	TP53	P04637	K132M	[L]Ubiquitylation
TP53	P04637	C238F	[L]Binding Site	TP53	P04637	K132Q	[L]Ubiquitylation
TP53	P04637	C242F	[L]Binding Site	TP53	P04637	K139N	[L]Ubiquitylation
TP53	P04637	R248L	[L]Binding Site	TP53	P04637	K132T	[L]Ubiquitylation
TP53	P04637	S241F	[L]Binding Site	TP53	P04637	K164E	[L]Acetylation
TP53	P04637	C242Y	[L]Binding Site	TP53	P04637	R273C	[L]Binding Site
TP53	P04637	C238Y	[L]Binding Site	TP53	P04637	R248Q	[L]Binding Site
TP53	P04637	R280K	[L]Binding Site	TP53	P04637	R248W	[L]Binding Site

Key pfsSNVs identification criteria: gene present in the list of 260 significantly mutated gene (SMG) set; gene present in the list of 573 cancer gene consensus (CGC) gene set; pfsSNV either exists across multiple cancer types or significantly associates with specific cancer type.
