# Supplementary material for: Distribution bias analysis of germline and somatic single-nucleotide variations that impact protein functional site and neighboring amino acids
Source: Sci Rep. 2017 Feb 8;7:42169. doi: 10.1038/srep42169 (PMC5296879; doi:10.1038/srep42169)
Supplement: Supplementary Figures [file srep42169-s1.pdf]

Distribution bias analysis of germline and somatic single-nucleotide variations that impact protein functional site and neighboring amino acids

Yang Pan[1]†, Cheng Yan[1]†, Yu Hu[1], Yu Fan[1], Qing Pan[2], Quan Wan[1], John Torcivia-Rodriguez[1] and Raja Mazumder[1,3]

[1]The Department of Biochemistry & Molecular Medicine, The George Washington University Medical Center, Washington, DC 20037, United States of America

[2]The Department of Statistics, The George Washington University, Washington, DC 20037, United States of America

[3]McCormick Genomic and Proteomic Center, The George Washington University, Washington, DC 20037, United States of America

†Y.Pan and C.Yan contributed equally to this article

*Corresponding author

Supplementary Figure Legend

SupFig1: Kaplan-Meier plot of pancreatic cancer patient survival based on the existence of MED16-H449Q-Phosphorylation. X-axis indicate days of survival and Y-axis indicates survival probability. Red and blue lines indicate survival time of pancreatic cancer patients with and without such mutation respectively. Log-rank test shows that, comparing with patients with MED16-H449Q-Phosphorylation, patients without this pfsSNV survive significantly longer with adjusted p-value of 0.05. The hazard ratio is 2.37

SupFig2: Kaplan-Meier plot of colon cancer patient survival based on the existence of PCDHA7-L352I-Phosphorylation. X-axis indicate days of survival and Y-axis indicates survival probability. Red and blue lines indicate survival time of pancreatic cancer patients with and without such mutation respectively. Log-rank test shows that, comparing with patients with PCDHA7-L352I-Phosphorylation, patients without this pfsSNV survive significantly longer with adjusted p-value of 0.05. The hazard ratio is 2.45

Supplementary Table Legend

SupTab1: Ratio of single nucleotide variations (SNVs) on protein functional site. For each mutation type (non-synonymous somatic, synonymous somatic, non-synonymous germline and synonymous germline), and its ratio was calculated separately.

SupTab2: Single nucleotide variations (SNVs) occurrence on protein functional site

region. For each mutation type, its occurrence on each amino acid position between 20 amino acid upstream / downstream of protein functional site was calculated to see if there is a statistical difference between the SNV occurrence on protein functional site and on neighboring region.

SupTab3: Amino acid type based single nucleotide variations (SNVs) occurrence on protein functional site. For each mutation type, its expected occurrence on each amino acid type was calculated as well as its occurrence on that amino acid type which is a protein functional site. Binomial test was applied to define the statistical difference between expected and observed SNV occurrence.

SupTab4: pfsSNVs existing in multiple cancer types. All pfsSNVs existing across more than three cancer types are listed with corresponding cancer types and the protein functional site.

SupTab5: pfsSNVs enriched in patient with specific cancer type. Occurrence of both gain and loss of protein functional site SNVs enriched in specific cancer type are calculated from patient information in TCGA dataset. Binomial test was applied to calculate the significance of pfsSNVs in corresponding cancer type.

SupTab6: Statistics of survival analysis for key pfsSNVs that have hazard ratio larger than 1. These key pfsSNVs do not significantly affect patients' survival (ProbChiSq < 0.05).

Supplementary Figure 1: Kaplan-Meier plot of the pancreatic cancer patient survival based on the existence of MED16-H449Q-Phosphorylation

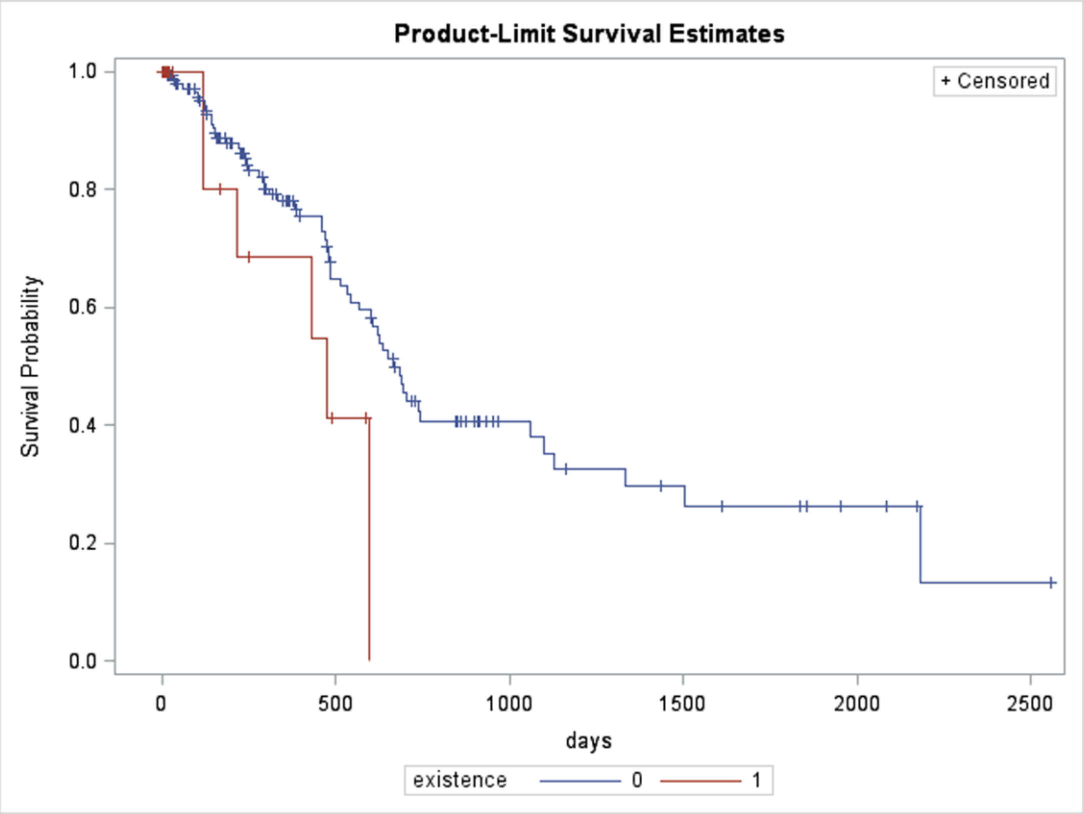

Test	Chi-Square	Pr > Chi-Square	Parameter	Parameter Estimate	Standard Error	Wald Chi-Square	Pr > ChiSq	Hazard Ratio
**Log-Rank**	3.7618	0.0524	Existence of mutation	0.86323	0.44826	3.7085	0.0541	2.371

Supplementary Figure 2： Kaplan-Meier plot of the colon cancer patient survival based on the existence of PCDHA7-L352I-Phosphorylation

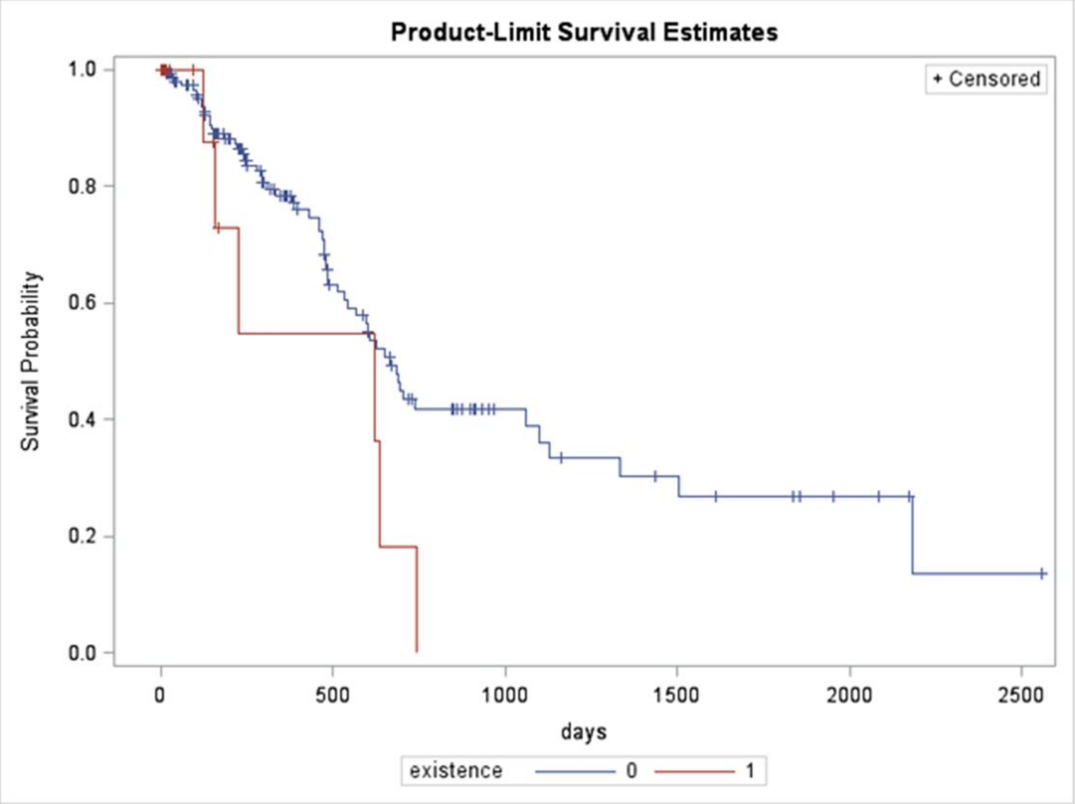

Test	Chi-Square	Pr > Chi-Square	Parameter	Parameter Estimate	Standard Error	Wald Chi-Square	Pr > ChiSq	Hazard Ratio
Log-Rank	3.4907	0.0617	Existence of mutation	0.8941	0.45966	3.7836	0.0518	2.445
